# Functional and surgical outcomes of ventral mesh rectopexy in male patients with rectal prolapse: a nationwide multicenter retrospective cohort study by the Pelvic Floor Working Group of the Turkish Society of Colon and Rectal Surgery (TSCRS)

**DOI:** 10.1007/s10151-026-03293-w

**Published:** 2026-04-03

**Authors:** F. A. Gultekin, B. Balcı, Y. Yıldırım, Ç. Arslan, N. F. Yaşar, T. Bisgin, A. Bayraktar, S. Zenger, N. Şengül, M. Çakır, O. Bozbıyık, R. Kozan, F. Y. Karakayalı, A. E. Canda, E. Öztürk, T. Bulut

**Affiliations:** 1 General Surgery Department, Zonguldak Bulent Ecevit University, School of Medicine, Zonguldak, Turkey; 2 General Surgery Department, ASV Yasam Hospital, Antalya, Turkey; 3 General Surgery Department, Florence Nightingale Hospital, Demiroglu Bilim University, School of Medicine, Istanbul, Turkey; 4 General Surgery Department, Istanbul Health and Technology University, School of Medicine, Istanbul, Turkey; 5 General Surgery Department, Eskisehir Osmangazi University, School of Medicine, Eskisehir, Turkey; 6 General Surgery Department, Dokuz Eylul University, School of Medicine, Izmir, Turkey; 7 General Surgery Department, Istanbul University, Istanbul Faculty of Medicine, Istanbul, Turkey; 8 General Surgery Department, VKV American Hospital, Istanbul, Turkey; 9 General Surgery Department, Bolu Abant Izzet Baysal University, School of Medicine, Bolu, Turkey; 10 General Surgery Department, Necmettin Erbakan University, School of Medicine, Konya, Turkey; 11 General Surgery Department, Ege University, School of Medicine, Izmir, Turkey; 12 General Surgery Department, Gazi University, School of Medicine, Ankara, Turkey; 13 General Surgery Department, Baskent University, School of Medicine, Istanbul, Turkey; 14 Private Clinic for Colorectal Surgery and Peritoneal Surface Malignancies, Izmir, Turkey; 15 General Surgery Department, Medicana Hospital, Bursa, Turkey

**Keywords:** Male sex, External rectal prolapse, Internal rectal prolapse, Fecal incontinence, Obstructive defecation syndrome, Ventral mesh rectopexy

## Abstract

**Background:**

The indications and outcomes of ventral mesh rectopexy (VMR) in the treatment of rectal prolapse in males have been minimally addressed in the literature. This study aimed to evaluate the demographics, clinical characteristics, and surgical outcomes associated with VMR in male patients.

**Methods:**

This nationwide retrospective cohort study included male patients who underwent VMR at centers performing pelvic floor surgery across Turkey. Clinical and surgical characteristics were obtained, and functional outcomes were evaluated using Cleveland Clinic Incontinence score (CCIS) and Altomare Obstructed Defecation Syndrome (ODS) scores at the pre- and postoperative periods.

**Results:**

Forty-one male patients (mean age, 45.4 years; mean BMI, 25.6 kg/m^2^) underwent VMR (2 open, 5 robotic, and 34 laparoscopic). The cohort comprised 11 patients with internal rectal prolapse (IRP) and 30 with external rectal prolapse (ERP). The overall complication rate was 17.1% (2.4% related to mesh), with a 12% recurrence rate. The median follow-up was 33 (6–127) months. The recurrence rate was 36.4% for IRP patients and 3.3% for ERP patients (*p* = 0.014). Significant improvements were observed in the CCIS (preoperative: 6.1 ± 4.8; postoperative: 2.8 ± 3.3, *p* = 0.005) and Altomare ODS (preoperative: 11.9 ± 4.6; postoperative: 7.26 ± 5.8, *p* < 0.001).

**Conclusions:**

In this nationwide retrospective cohort study, VMR was associated with favorable functional outcomes in males. Recurrence rates were significantly higher in those with internal rectal prolapse. As sexual function was not systematically assessed, prospective studies with standardized patient-reported outcomes are warranted.

## Introduction

External rectal prolapse (ERP) is the protrusion of the rectum through the anus during defecation, either partially or as full thickness [1], whereas internal rectal prolapse (IRP) refers to telescopic invagination of the rectum or anal canal without external protrusion [2]. Rectal prolapse severely impairs quality of life by causing fecal incontinence and obstructed defecation syndrome. Although the true incidence is unknown, male patients represent only 10% of the surgically treated patients [3]. Among surgical techniques, ventral mesh rectopexy (VMR) stands out in the treatment of ERP and high-grade IRP unresponsive to conservative therapy with low recurrence and de novo symptom rates [4].

However, performing VMR in the narrow pelvis of male patients with rectal prolapse carries a risk of autonomic nerve injury, which may result in sexual dysfunction [5]. In fact, a previously published consensus statement on VMR identified IRP as a relative contraindication for VMR [6]. Nevertheless, the number of studies evaluating the outcomes of VMR in males with ERP and IRP remains limited. Indeed, a recent systematic review of 28 studies [7] and meta-analysis including 8 studies [8] revealed that, after excluding overlapping data, only 186 of 2053 male patients with rectal prolapse underwent VMR. Of these, only two studies [9, 10] focused exclusively on male patients, with sample sizes of 52 and 68. These findings indicate a continuing need for studies evaluating the outcomes of VMR in males with rectal prolapse.

The Pelvic Floor Disorders Working Group (PFDWG) was established in 2023 with the support of the Turkish Society of Colon and Rectal Surgery (TSCRS). The group was formed to raise awareness about pelvic floor disorders among the public and surgeons, to organize educational meetings with theoretical and practical content, to promote multidisciplinary collaboration with other specialties in the treatment of pelvic floor disorders, and to conduct clinical research. The TSCRS-PFDWG consists of colorectal surgeons from academic and private hospitals in Turkey who perform high-volume pelvic floor surgery. One of the questions raised by the participants during the group’s educational meetings was about performing VMR in male patients with rectal prolapse. In light of these concerns and the limited literature on the topic, the TSCRS-PFDWG designed the present study to assess the functional and surgical outcomes of VMR in male patients with rectal prolapse.

## Methods

### Study design and data collection

This was a multicenter, retrospective observational study in which clinical data were extracted from institutional medical records and collected in a prospectively designed dataset. A committee composed of members of the TSCRS-PFDWG was assigned to develop the structured dataset form and to distribute it to the high-volume surgeons (at least 20 prolapse procedures per year) performing pelvic floor disorder surgery. Among the 20 surgeons invited, 11 had performed VMR on male patients with rectal prolapse. Submitted data from each surgeon were verified by the investigators of the study.

The study was approved by the Ethics Committee of Istanbul Medipol University (05/07/2024, E-10840098-202.3.02-3983) and was reported according to the STROBE guidelines [11].

### Inclusion criteria

The inclusion criteria were male sex, age > 16 years, diagnosis of internal rectal prolapse (Oxford grade III and IV) [12] and/or external rectal prolapse, and operation with VMR (open, laparoscopic, or robotic) between June 2014 and 2024 with at least 6 months of follow-up. Those who had obstructive defecation syndrome without rectal prolapse related to pelvic dyssynergia, slow-transit constipation, or Hirschsprung disease and who underwent other rectopexy techniques were excluded. In addition, patients who had inflammatory bowel disease and a history of pelvic radiotherapy were not included in this study. Patients whose baseline and 6-month Cleveland Clinic Incontinence Score (CCIS) [13] and Altomare Obstructed Defecation Syndrome (ODS) [14] score were not recorded, and those who did not attend the 6-month clinic visit in person were not included in the analysis.

### Study variables and outcomes

Demographic and clinical characteristics, including age, body mass index (BMI), smoking status, marital status, sexual orientation, and comorbidities, were recorded. Primary presenting symptoms, as well as the presence of anal-retentive behavioral disorders [15] and associated psychiatric conditions, were evaluated via relevant ICD codes and hospital records. Details of diagnostic investigations performed to identify underlying or concomitant pathologies were also retrieved.

Operative data included the type of mesh used (biologic, synthetic, or composite) and the fixation technique, either to the rectum and/or levator ani muscle or to the sacral promontory via tacks or sutures. Postoperative 30-day complications and recurrence rates were documented. Postoperative complications were categorized and analyzed using the Clavien-Dindo classification system [16].

Functional outcomes were assessed using the CCIS and the Altomare ODS score, both preoperatively and at 6 months postoperatively. For patients lacking these records, data were collected retrospectively through telephone interviews with those who were reachable.

Owing to the limited number of patients, validated sexual function questionnaires were not included as a formal outcome in this study. However, patients were asked a binary “yes/no” question regarding the presence of any negative impact on sexual function following surgery. Persistent dysfunction lasting > 6 months was classified as sexual dysfunction.

Although follow-up protocols vary across centers, all included patients were required to have an outpatient visit at 6 months (± 1 month) and a repeat evaluation using the same preoperative functional scoring systems. Beyond the 6-month timepoint, patients were typically evaluated on a symptom-driven basis. All patients who attended the 6-month follow-up visit and were included in the analysis were subsequently contacted again in May 2025 during the data collection period to inquire about current symptoms. No suspected recurrences were identified during this telephone follow-up.

Recurrence assessment for ERP was performed via physical examination in the prone or commode position. Patients who were symptomatic but had normal physical examination findings underwent magnetic resonance (MR) defecography to evaluate for recurrence.

### Statistical analysis

The statistical software package SPSS 22.0 for Windows^®^ (SPSS Inc., Chicago, IL) was used. Continuous variables are reported as means with standard deviations if normally distributed or medians with ranges if abnormally distributed. Categorical variables are presented as frequencies with percentages. Continuous variables were compared using the chi-square test. The differences between the pre- and postoperative 6-month CCIS and Altomare ODS score were analyzed using the Wilcoxon signed-rank test; *p* < 0.05 was considered significant.

## Results

Forty-one male patients (mean age, 45.4 ± 16.5 years; mean BMI, 25.6 ± 3.9 kg/m^2^) with rectal prolapse underwent VMR. A history of psychiatric disorders was present in nine patients (22%). In the study cohort, eight patients (19.5%) had previously undergone proctologic or pelvic floor surgery: Longo procedure (*n* = 4), suture rectopexy (*n* = 1), ventral mesh rectopexy (*n* = 1), resection rectopexy (*n* = 1), and Delorme procedure (*n* = 1). The most common primary symptoms included prolapse in 25 patients (61%) and constipation in 10 patients (24.4%). The median duration of symptoms in patients was 24 (1–479) months. Anismus and chronic pelvic pain were detected in 17.1% and 24.2% of patients, respectively. Details of the demographic and clinical characteristics are given in Table 1.

**Table 1:** Demographic and clinical characteristics of patients

	Total *n* = 41	(%)
Age (years, mean ± SD)	45.4 ± 16.5	
BMI (kg/m^2^, mean ± SD)	25.6 ± 3.9	
Smoking (+)	16	39%
Comorbidity	13	31.7%
Single marital status	12	29%
Sexual orientation		
Heterosexual	38	92.7%
LGBTQ +	3	7.3%
Psychiatric disease	9	22%
Anal retentive behavior	11	26.8%
Previous proctologic or pelvic floor surgery	8	19.5%
Duration of the symptoms (months, median, range)	24	1–479
Primary complaint		
Rectal prolapse	25	61%
Constipation and/or ODS	10	24.4%
Proctologic complaints	4	9.8%
Fecal incontinence	2	4.9%
Bowel movements (per week, median, range)	7	1–40
Anismus	7	17.1%
Solitary rectal ulcer	4	9.8%
Chronic pelvic pain	10	24.2%
Urinary symptoms	13	31.7%
Sexual dysfunction	4	9.8%

LGBTQ +: lesbian, gay, bisexual, transgender, queer, plus

The frequency of diagnostic test utilization is summarized in Table 2. The most used test was MR defecography, which was performed in all patients with IRP and ten patients (33%) with ERP. MR defecography demonstrated Oxford grade IV IRP in eight patients and Oxford grade III IRP in three patients. No concomitant enterocoele or peritoneocele was identified in any patient in the study cohort. The second most frequently used test was the balloon expulsion test, which was performed in eight patients across the entire cohort. Despite an anismus rate of 17.1% being reported, pelvic floor physiotherapy was performed in only 7.3% of the overall cohort and in 27% of patients with IRP.

**Table 2:** Preoperative work-up and pelvic floor physiotherapy (PFP)

	Total *n* = 41	IRP *n* = 11	ERP *n* = 30
MR defecography	20 (48.8%)	11 (100%)	9 (30%)
Conventional defecography	1 (2.4%)	–	1 (3.3%)
Colonic transit time	1 (2.4%)	1 (1%)	–
Anal manometry	3 (7.3%)	–	3 (9.9%)
Balloon expulsion test	5 (12.2%)	3 (27%)	2 (6.6%)
Preoperative PFP	3 (7.3%)	3 (27%)	–

*IRP* internal rectal prolapse, *ERP* external rectal prolapse, *MR* magnetic resonance, *QoL* quality of life, *PFP* pelvic floor physiotherapy

The surgical approach included laparoscopic surgery in 34 patients (82.9%), followed by robotic surgery in 5 patients (12.2%) and open surgery in 2 patients (4.9%) (Table 3). The mean operative time for the laparoscopic approach was 128 ± 35.1 min. The majority of patients received a synthetic polypropylene mesh (73.2%), while composite and biologic meshes were used in 24.4% and 2.4% of cases. Pelvic fixation was performed on the rectum alone in 70.7% of patients, on the levators alone in 19.5%, and on both sites in 9.8%. Absorbable sutures were the most commonly used pelvic fixation material (41.5%), whereas sacral fixation was primarily achieved with non-absorbable tacks (34.1%) or sutures (29.3%). Other combinations of fixation materials were used less frequently (Table 3).

**Table 3:** Surgical characteristics of patients and details of surgical techniques

	Total *n* = 41	(%)
Etiology		
IRP	11	26.8%
ERP	30	73.2%
0–5 cm	16	55.2%
5–10 cm	11	37.9%
> 10 cm	2	6.9%
Presentation		
Primary	33	80.5%
Recurrent	8	19.5%
Surgery		
Open	2	4.9%
Laparoscopic	34	82.9%
Robotic	5	12.2%
Operative time (min, mean ± SD)		
Open	150 ± 0	
Laparoscopic	128 ± 35.1	
Robotic	140 ± 7	
Mesh material		
Polypropylene	30	73.2%
Composite	10	24.4%
Biologic	1	2.4%
Pelvic fixation		
Rectum only	29	70.7%
Levators only	8	19.5%
Rectum and levators	4	9.8%
Pelvic fixation material		
Absorbable sutures	17	41.5%
Absorbable tacks	7	17.1%
Absorbable sutures and absorbable tacks	7	17.1%
Non-absorbable sutures	6	14.6%
Slow absorbable sutures	4	9.8%
Sacral fixation material		
Non-absorbable tack	14	34.1%
Non-absorbable suture	12	29.3%
Absorbable suture + non-absorbable tack	8	19.5%
Non-absorbable suture + non-absorbable tack	7	17.1%

Postoperative complications were identified in 17.1% of patients (*n* = 7) within the first 30 days following surgery. According to the Clavien-Dindo classification system, five complications were classified as grade I, and two complications were classified as grade IIa (Table 4). The complication rate was 36.4% in the IRP group and 10% in the ERP group; however, this difference did not reach statistical significance (*p* = 0.069). Mortality was not reported in any of the patients during the study period.

**Table 4:** Postoperative complications, recurrence, and follow-up

	Total *n* = 41	IRP *n* = 11		ERP *n* = 30	*p*
Follow-up, months, median (range)	33 (6–127)				
Mortality, *n* (%)	0				
Complications, *n* (%)	7 (17.1)	4 (36.4)	3 (10)		0.069
Clavien-Dindo classification					
Grade I (*n*)	5	3	2		
Grade IIa (*n*)	2	1	1		
Recurrence, *n* (%)	5 (12)	4 (36.4)	1 (3.3)		**0.014**
Time-to-recurrence, months, median (range)	24 (5–60)	24 (6–60)	5 (5–60)		–

*IRP* internal rectal prolapse, *ERP* external rectal prolapse

The mean preoperative CCIS and Altomare ODS scores were 5.6 ± 5.2 and 10.6 ± 4.5, respectively, in patients with IRP and 7.0 ± 3.7 and 12.7 ± 6.3, respectively, in those with ERP (Table 5). The functional assessments at 6 months postoperatively demonstrated a statistically significant improvement in both the CCIS and the Altomare ODS score in both groups.

**Table 5:** Functional outcomes of the study cohort

	Total *n* = 41	IRP *n* = 11	ERP *n* = 30	
CCIS, mean ± SD		*p* = 0.005*	*p* < 0.001*	
Preoperative	6.1 ± 4.8	5.6 ± 5.2	7 ± 3.7	0.424
Postoperative	2.8 ± 3	3.8 ± 3.6	2.3 ± 2.6	0.231
Altomare ODS score, mean ± SD		*p* = 0.005*	*p* < 0.001*	
Preoperative	11.9 ± 4.6	10.6 ± 4.5	12.7 ± 6.3	0.212
Postoperative	7.26 ± 5.8	6.3 ± 5.5	7.8 ± 5.9	0.489

*IRP* internal rectal prolapse, *ERP* external rectal prolapse, *CCIS* Cleveland Clinic Incontinence Score, *ODS* obstructed defecation syndrome, *SD* standard deviation

*Wilcoxon signed-rank test to compare the CCIS and Altomare ODS scores between pre- and postoperative measurements

The median follow-up duration was 33 (range 6–127) months. The recurrence rates were 36.4% in the IRP group and 3.3% in the ERP group, with the difference reaching statistical significance (*p* = 0.014). Among patients who experienced recurrence, operative characteristics were further examined and are summarized in Table 6. In the recurrence cohort (*n* = 5), all patients underwent laparoscopic VMR using polypropylene mesh. Four of the five recurrences occurred in IRP patients. Rectal fixation with absorbable sutures was the predominant pelvic fixation method, and non-absorbable tacks were used for sacral fixation in most cases. Despite similar operative materials and techniques, recurrence was more frequently observed among IRP patients (*n* = 4).

**Table 6:** Operative characteristics of recurrent cases following VMR in male patients with ERP and IRP

	Total (*n* = 5)	IRP (*n* = 4)	ERP (*n* = 1)
Surgery *n* (%)			
Open			
Laparoscopic	5	4 (80)	1 (20)
Robotic			
Mesh material *n* (%)			
Polypropylene	5	4 (80)	1 (20)
Composite			
Biologic			
Pelvic fixation *n* (%)			
Rectum only	4 (80)	4 (80)	
Levators only			
Rectum and levators	1(20)		1 (20)
Pelvic fixation material *n* (%)			
Absorbable sutures	4(80)	4 (80)	
Absorbable tacks	1 (20)		1(20)
Absorbable sutures and absorbable tacks			
Non-absorbable sutures			
Slow absorbable sutures			
Sacral fixation material *n* (%)			
Non-absorbable tack	4 (80)	3 (60)	1 (20)
Non-absorbable suture	1 (20)	1 (20)	
Absorbable suture + non-absorbable tack			
Non-absorbable suture + non-absorbable tack			

*IRP* internal rectal prolapse, *ERP* external rectal prolapse

## Discussion

This nationwide, multicenter study demonstrated that VMR, when performed by high-volume pelvic floor surgeons, is a safe and effective procedure in male patients, as evidenced by low morbidity and mortality rates and significantly improved fecal incontinence and ODS scores at 6 months postoperatively. However, the study presents noteworthy findings regarding recurrence following VMR in male patients. While the recurrence rates associated with ERP were lower than those reported in the literature, the median time to recurrence was 5 months. In contrast, among 11 patients with IRP, recurrence occurred in 4 patients (36.4%) at a median of 24 months. Although the relatively small sample size—particularly within the IRP subgroup—and the lack of a standardized postoperative sexual function assessment due to inconsistent data collection across participating centers limit the generalizability and depth of the findings, this study nonetheless provides a valuable contribution to the limited body of literature. To date, only two studies have exclusively reported outcomes of VMR in male patients [9, 10].

Male patients with rectal prolapse represent only a small subset of the overall rectal prolapse population, with a reported female-to-male ratio of approximately 6:1 [3, 17]. Most published data on surgical outcomes do not differ between sexes, and results are commonly reported irrespective of sex. However, male rectal prolapse patients should be considered a distinct group because of differences in pelvic anatomy, the tendency for the condition to occur at a younger age, and the involvement of potentially different pathophysiologic mechanisms, such as chronic constipation, excessive straining, and behavioral disorders [3]. A systematic review and meta-analysis published in 2023 and 2024 on surgical outcomes in male rectal prolapse highlighted the scarcity of studies specifically designed to address sex-based differences [7, 8]. Among the studies included in these reviews, only two evaluated outcomes of VMR in male patients: one involving 52 ERP cases [9] and the other comprising 18 ERP and 50 IRP cases [10]. Compared with previous reports, the present study includes a relatively larger cohort of male patients, comprising 11 with IRP and 30 with ERP.

In line with previous reports [3, 17], our study also revealed that rectal prolapse in males tends to occur at a younger age, with a mean of 45.4 ± 16.5 years. Constipation, anismus, and chronic pelvic pain were the most reported symptoms associated with rectal prolapse. Psychiatric comorbidities were identified in 22% of the patients, and anal-retentive behavior was observed in 26.8%. The median duration of symptoms before presentation was 24 months, with the time from symptom onset to seeking medical attention ranging from 1 to 479 months. The co-occurrence of long-standing constipation and/or ODS with psychiatric and behavioral disorders in male patients with rectal prolapse has been described [18, 19], and a similar pattern was observed in our cohort. Chronic pelvic pain, which was present in 24% of the patients in this study, may have a psychologic origin; however, it remains a poorly understood symptom. Previous studies have reported that the presence of both IRP and ODS increases the likelihood of pelvic pain up to 73% [20]. The underlying mechanism of pain in these patients may involve a combination of levator diastasis-related ischemia and pudendal nerve stretching.

Although VMR is technically more challenging in male patients, only minor complications were observed within the first 30 postoperative days in this study (*n* = 7, 17.1%). The complication rates reported here are comparable to those reported in previous studies evaluating VMR outcomes in male rectal prolapse patients, such as Rautio et al. (17.3%) and Owais et al. (10%) [9, 10]. Notably, the complication rates were greater in patients who underwent VMR for IRP than in those for ERP, although the difference was not statistically significant.

Despite growing concerns regarding the use of synthetic mesh, the complication rates reported by experienced surgeons remain low, ranging from 0.7 to 2.4% with the use of either biologic or synthetic mesh [21]. Similarly, no mesh-related complications were observed in the present study, in which all VMR procedures were performed by high-volume pelvic floor surgeons.

The median follow-up duration in our study was 33 months. Although this duration is somewhat shorter than the follow-up durations reported by Rautio et al. (56.1 months) and Hu et al. (48.5 months) [9, 22], it remains within a reasonable range. Notably, 70% of our patients (*n* = 29) completed at least 2 years of follow-up, and patients with a follow-up duration of < 6 months were excluded from our analysis. In comparison, Owais et al. reported a longer mean follow-up of 42 months, although only 12 patients completed the full follow-up period [10].

Several studies have reported that VMR may offer greater improvement in terms of fecal incontinence than other surgical approaches [23, 24], but evidence specific to male patients remains limited. Owais et al. reported significant improvements in functional outcomes and quality of life following VMR in male patients with both IRP and ERP, without differentiating subtypes [10]. In contrast, Hu et al. reported that VMR was associated with a reduction in constipation scores but a worsening of fecal incontinence in male patients with ERP [22]. In our study, VMR led to improvements in both constipation and incontinence scores in patients with IRP and ERP. In the study by Rautio et al., although preoperative functional data were lacking, postoperative assessments indicated better continence and defecation outcomes in patients < 40 years of age, whereas older patients reported higher symptom scores [9].

One of the major limitations of this study is the absence of a standardized, validated assessment of sexual function across participating centers. Although postoperative sexual function was recorded using a binary (yes/no) patient-reported outcome, this method is insufficient to characterize the severity, nature, or impact of dysfunction. This limitation is particularly important in male patients VMR, where anterior pelvic dissection poses a potential risk of autonomic nerve injury (5), especially within the confines of the narrow male pelvis (5). The lack of detailed functional data restricts the ability to meaningfully evaluate this critical outcome. Future prospective studies should incorporate validated instruments such as the International Index of Erectile Function (IIEF-5) or male-adapted versions of the Female Sexual Function Index (FSFI-M) to allow a more nuanced and reproducible evaluation of sexual health after VMR. These tools are essential not only for outcome measurement but also for guiding preoperative counseling and shared decision-making.

Male sex has been consistently identified as a potential risk factor for recurrence [25]. Emile et al. reported a recurrence rate of 11.2% in male ERP patients, whereas Fagan et al. noted a broader range (0–34%) after abdominal rectal prolapse surgery in males [7, 8]. Moreover, a recent systematic review reported recurrence rates of 0–18.8%, regardless of sex [25]. In our cohort, recurrence occurred in 12% of patients, with a median time to recurrence of 24 months. Although our follow-up duration appears adequate for assessing mid-term outcomes, previous studies have shown that recurrences can occur beyond the 3rd postoperative year [26]. Therefore, our recurrence data may underestimate the true long-term failure rate, particularly in patients with IRP, and should be interpreted with caution.

Several factors may contribute to recurrence after VMR, including mesh type, fixation method, and patient-related factors such as age, obesity, and connective tissue disorders [27]. In male patients, narrow pelvic anatomy may limit adequate anterior dissection and mesh placement, potentially increasing recurrence. However, in our cohort, operative characteristics were comparable between recurrent and non-recurrent cases, suggesting that technical variables alone do not account for failure. IRP patients exhibited higher recurrence rates than ERP patients, consistent with previous reports [10]. Emile et al. recommended evaluating IRP and ERP as distinct entities due to differences in rectal wall biomechanics [28]. Moreover, IRP is often associated with pelvic floor dysfunctions such as rectocele or dyssynergic defecation, which may not be fully addressed by VMR.

More recently, Cooper et al. proposed that the anatomical location of the prolapse’s leading point, as visualized on defecography, may be a key determinant of recurrence risk [29]. According to this concept, ventral mesh rectopexy may be preferable in high take-off prolapse, while perineal procedures (e.g., Delorme or Altemeier) may be more suitable in low take-off prolapses to minimize recurrence. In our study, all IRP patients underwent VMR for Oxford grade III–IV prolapse; however, the leading point of prolapse (i.e., high vs. low take-off) was not systematically assessed. This likely reflects the novelty of the concept at the time of data collection. The relatively high recurrence rate observed in IRP patients may thus be partially explained by the unselected application of VMR in cases with potentially low take-off prolapse, where a perineal approach might have been more appropriate.

These observations underscore that IRP in male patients should be approached as a distinct condition with a higher risk of recurrence following VMR. Given current consensus guidelines that consider IRP a relative contraindication for ventral mesh rectopexy, careful patient selection and individualized surgical planning are essential in this subgroup.

Although these clinical patterns suggest important directions for patient selection, our ability to formally identify independent risk factors was limited. While multivariate analysis would have been useful for identifying predictors of recurrence, it was not performed in this study because of the small sample size and the limited number of recurrences, which would have resulted in insufficient statistical power and a high risk of model overfitting. Therefore, the findings—particularly those regarding IRP—should be interpreted with caution. Future research incorporating standardized defecographic assessment and larger, prospectively collected patient cohorts will be essential to refine patient selection and improve long-term outcomes.

This study has several limitations that warrant consideration. First, the retrospective design inherently limits the ability to establish causal relationships and introduces potential selection and information bias. Although data were retrieved from a prospectively maintained registry, missing or inconsistent entries—particularly regarding long-term outcomes and sexual function—may have influenced the results. The absence of a validated, standardized assessment tool for sexual function across participating centers represents a notable limitation of this study. Reliance on a binary, patient-reported yes/no item precluded a nuanced evaluation of sexual dysfunction following VMR and limited the interpretability of this important outcome. Additionally, in a subset of patients, follow-up data were obtained via telephone interviews, which may be subject to recall bias and lack the precision of standardized in-person assessments. The relatively small overall sample size, particularly in the IRP subgroup, limits the statistical power of the study and increases the risk of type II error; therefore, some observed differences may not have reached statistical significance despite potential clinical relevance. The follow-up duration, while adequate for mid-term evaluation, may be insufficient to capture all recurrence events, particularly given that some recurrences occurred beyond 24 months. Therefore, the true recurrence rate—especially in patients with IRP—may be underestimated. Additionally, heterogeneity in operative technique, mesh selection, and fixation methods across centers may have influenced surgical outcomes. Finally, as all cases were performed in high-volume pelvic floor units, the generalizability of these findings to lower-volume or less specialized settings may be limited.

In conclusion, this nationwide, multicenter study presents a relatively sizable cohort focusing exclusively on male patients who underwent VMR for rectal prolapse. By distinguishing outcomes between ERP and IRP subtypes and incorporating detailed functional assessments, valuable insights into a frequently overlooked patient population can be obtained. The strength of this study lies in its collaboration among high-volume pelvic floor surgeons and the use of a standardized national registry. Future prospective studies with longer follow-up, sexual function metrics, and stratified analysis by prolapse type are needed to optimize patient selection and refine surgical strategies for the management of rectal prolapse in males.

## Data Availability

The datasets generated during and/or analysed during the current study are available from the corresponding author on reasonable request.
